# Retraction: Anti-neuroinflammatory and neuroprotective potential of *Cissus tuberosa* ethanol extract in Parkinson’s disease model through the modulation of neuroinflammatory markers

**DOI:** 10.1371/journal.pone.0360896

**Published:** 2026-10-20

**Authors:** The PLOS One Editors

Following the publication of this article [1] concerns were raised about results published in Tables 6-7 and unpublished image data provided with the original submission of [1]. Specifically,

- In Tables 6 and 7, the reported values for the standard error of the mean appear unusually repetitive.
- The following panels appear similar, despite being used to represent different experimental conditions:
  - C Heart in unpublished material submitted for [1] and Fig 2 CG:Heart of [2]
  - C Liver in unpublished material submitted for [1] and Fig 2:TR Liver of [2]
- The CG panel in unpublished material submitted for [1] and the Fig 3 CG panel of [3, retracted in 4]

Regarding the concerns with similarities between the unpublished material for this article and the material published in [3, retracted in 4], the corresponding author stated that errors were made during the figure preparation of the images presented in the original version of this manuscript, which were corrected in subsequent revisions. Regarding the concerns with similarities between the unpublished material in this article and the material published in [2], the corresponding author stated that the material in [2] was used without the data owner’s authorization.

The corresponding author provided the individual-level data underlying the graphs and tables presented in this article. Editorial assessment of these data raised additional concerns about the integrity and reliability of these data.

In light of the concerns observed with the individual-level data underlying this study, the *PLOS One* Editors retract this article.

ZC and MA responded but did not clarify either agreement or disagreement. MS, ASehar, RS, HT, SAB, and ASethi either did not respond directly or could not be reached.
